# Gut Microbiota Deficiency Exacerbates Liver Injury in Bile Duct Ligated Mice via Inflammation and Lipid Metabolism

**DOI:** 10.3390/ijms24043180

**Published:** 2023-02-06

**Authors:** Xueqian Zhou, Xiaoxun Zhang, Nan Zhao, Liangjun Zhang, Wen Qiu, Chunwei Song, Jin Chai, Shiying Cai, Wensheng Chen

**Affiliations:** 1 Cholestatic Liver Diseases Center, Department of Gastroenterology, Southwest Hospital, Third Military Medical University (Army Medical University), Chongqing 400038, China; 2 The Liver Center, Yale University School of Medicine, 333 Cedar Street, New Haven, CT 06520, USA

**Keywords:** cholestasis, gut microbiota, transcriptome, liver injury, biliary ligation

## Abstract

Bile components play a critical role in maintaining gut microbiota homeostasis. In cholestasis, bile secretion is impaired, leading to liver injury. However, it remains to be elucidated whether gut microbiota plays a role in cholestatic liver injury. Here, we performed a sham operation and bile duct ligation (BDL) in antibiotic-induced microbiome depleted (AIMD) mice and assessed liver injury and fecal microbiota composition in these mice. Significant reductions in gut microbiota richness and diversity were found in AIMD-sham mice when compared to sham controls. Three-day BDL leads to great elevation of plasma ALT, ALP, total bile acids, and bilirubin where reduced diversity of the gut microbiota was also found. AIMD further aggravated cholestatic liver injury evidenced by significantly higher levels of plasma ALT and ALP, associated with further reduced diversity and increased Gram-negative bacteria in gut microbiota. Further analyses revealed increased levels of LPS in the plasma of AIMD-BDL mice where elevated expression of inflammatory genes and decreased expression of hepatic detoxification enzymes were also found in liver when compared to the BDL group. These findings indicate that gut microbiota plays a critical role in cholestatic liver injury. Maintaining its homeostasis may alleviate liver injury in patients with cholestasis.

## 1. Introduction

Bile, formed in the liver and excreted into the gut, plays an important role in maintaining the homeostasis of gut microbiota. Disrupted homeostasis of gut microbiota has been associated with some liver diseases, including hepatocellular carcinoma, autoimmune hepatitis (AIH), nonalcoholic fatty liver disease (NAFLD), etc., indicating there is a reciprocal relationship between liver health and gut microbiota homeostasis. Recent studies revealed that altered gut microbiota was found in patients with primary biliary cholangitis, suggesting that gut microbiota plays a role in the pathogenesis of this cholestatic liver injury [1]. However, how gut microbiota may affect liver health and diseases remains elusive.

Reduced gut microbial diversity is now known to be associated with a variety of diseases such as: depression, autism, obesity, hypercholesterolemia, diabetes, inflammatory bowel disease, allergies, polycystic ovary syndrome, nonalcoholic fatty liver disease, colon cancer, and liver cancer [2,3,4,5,6,7,8,9]. In animal experiments, it is suggested that some microbial community alterations can directly lead to disease states [10]. One of the most famous experiments was that germ-free mice transplanted with microbial colonies from obese mice became obese more rapidly than germ-free mice transplanted with microbial colonies from lean mice [11,12]. Sunny H. Wong et al. used fecal transplantation in the field of oncology research and demonstrated that colon cancer patients’ colonies transplanted to germ-free animals could activate inflammatory and pro-oncogenic molecular pathways and promote intestinal tumorigenesis [13]. The above findings suggest that specific components of the microflora may have a major influence [10].

Antibiotic-induced microbiome depletion (AIMD) animals and germ-free animals are widely used to study the effects of gut microbiota on host physiology and disease. In the AIMD model, antibiotics reduce gut microbiota diversity to mimic a state of imbalance. AIMD can lead to significant changes in the microbiota metabolites, which affect gut signaling [14]. For example, the level of short-chain fatty acids (SCFAs) in the guts of AIMD mice decreases, and enterocytes meet their metabolic needs through enhanced glucose utilization [14,15]. This leads to a decrease in serum glucose, an increase in insulin sensitivity, and an increase in liver gluconeogenesis, thereby affecting systemic glucose homeostasis [14]. In addition, elevated serum levels of tauro-β-muricholic acid, which inhibits FXR activity in hepatocytes due to more gut uptake of tauro-β-muricholic acid than taurocholate acid, leads to increased hepatic synthesis of bile acids and alters the bile acid metabolite pool [14]. Tauroursodeoxycholic acid, due to its cytoprotective effect, has shown potential therapeutic effects in many disease models, such as diabetes, obesity, and neurodegenerative diseases, in addition to being used to treat liver diseases [16]. Interestingly, tauroursodeoxycholic acid was significantly increased in serum of the AIMD model [14]. These reports suggest that AIMD-induced changes in bile acid metabolism may affect the host in a variety of ways, but the effects of AIMD on mouse bile duct ligation (BDL) models was not clear.

In this study, we conducted biliary ligation surgery on the AIMD model, evaluated serum markers of liver injury and histological changes after BDL in the AIMD model, examined fecal microbiota composition, and performed transcriptome sequencing of liver tissue. By exploring the effect of AIMD on obstructive cholestatic liver injury, our study may provide new evidence for clarifying the mechanism of gut microbiota in the occurrence and development of cholestatic liver disease.

## 2. Results

### 2.1. AIMD Aggravates Liver Injury after BDL

**Figure 1:**
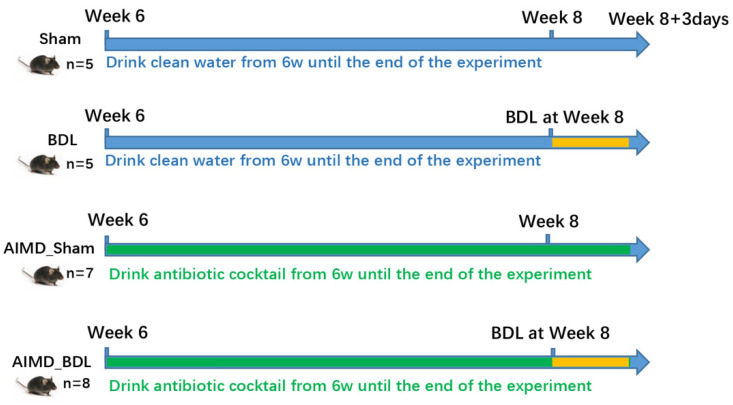
Experimental flowchart. The C57BL/6J male mice were divided into four groups (Sham group, AIMD_Sham group, BDL group, and AIMD_BDL group) using a random assignment table. The mixed antibiotics were added to the drinking water of AIMD_Sham and AIMD_BDL groups at week 6. All groups completed the corresponding Sham surgery or BDL surgery at week 8. Three days after completion of the sham ligation and bile duct ligation procedures, all groups of mice were weighed and euthanized.

The experimental flow diagram of each group is shown in Figure 1. The six-week-old C57BL/6J male mice were randomly divided into four groups (Sham, AIMD_Sham, BDL, and AIMD_BDL group). As shown in Table 1, there was no significant difference in serum liver-function markers between the Sham group and AIMD_Sham group, indicating that the dose of mixed antibiotics in the drinking water was safe for mice. However, BDL significantly increased serum ALT, AST, ALP, and TBA in these mice, and feeding with antibiotic water further increased these liver function parameters in the AIMD_BDL group when compared with the BDL group, suggesting that AIMD exacerbated the liver injury caused by BDL for 3 days. As shown in Figure S1, HE staining of liver tissues showed significantly higher scores for necrosis in the AIMD_BDL mice compared with the BDL mice.

### 2.2. AIMD Alters Gut Microbiota Alpha Diversity in Mice

**Figure 2:**
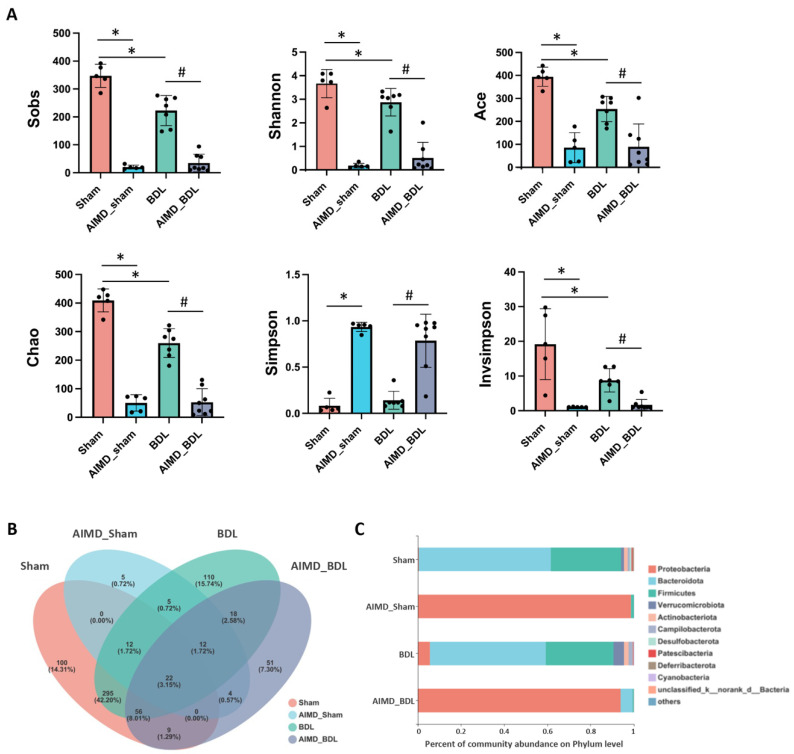
(**A**) Within-sample α-diversity analysis of gut microbiota, including Sobs, Shannon, Ace, Chao, Invsimpson, and Simpson in four groups. Sham, *n* = 5; AIMD_Sham, *n* = 5; BDL, *n* = 7; AIMD_BDL groups, *n* = 8. * *p* < 0.05 vs. Sham group; # *p* < 0.05 vs. BDL group. (**B**) Venn diagrams of OTUs for four groups. (**C**) The composition of the four groups at the phylum level.

After 16S rRNA sequencing of 25 fecal samples using the Miseq PE300 platform, 1,163,537 high-quality sequences were obtained after merging and filtering, and the sequencing details for each sample is shown in Table S1. We used the Good’s coverage index to analyze the species coverage of all samples and as shown in Table S2, the coverage of all samples was greater than 99%. To understand why worse liver injury was seen in AIMD_BDL group, we analyzed the gut microbiota in these animals. Compared with the Sham group, the AIMD_Sham group showed significant changes in various indices reflecting microbial abundance and diversity. When comparing the BDL group with the Sham group, we found that both the richness indexed by Sobs, Ace, Chao, and community diversity indexed by Shannon, Invsimpson were significantly lower in the BDL group. Interestingly, the Sobs, Shannon, Ace, Chao, and Invsimpson indices of the AIMD_BDL group were further significantly lower than those of the BDL group (Figure 2A).

We identified 1104 OTUs from 25 fecal samples, which can be divided into 20 phyla, 43 classes, 100 orders, 160 families, 302 genera, and 499 species. Venn diagrams showed there were 34 OTUs in both Sham and AIMD_Sham groups, 460 OTUs unique to the Sham group, and 26 OTUs unique to the AIMD_Sham group, indicating that the use of antibiotics impaired gut microbiota diversity (Figure 2B).

**Figure 3:**
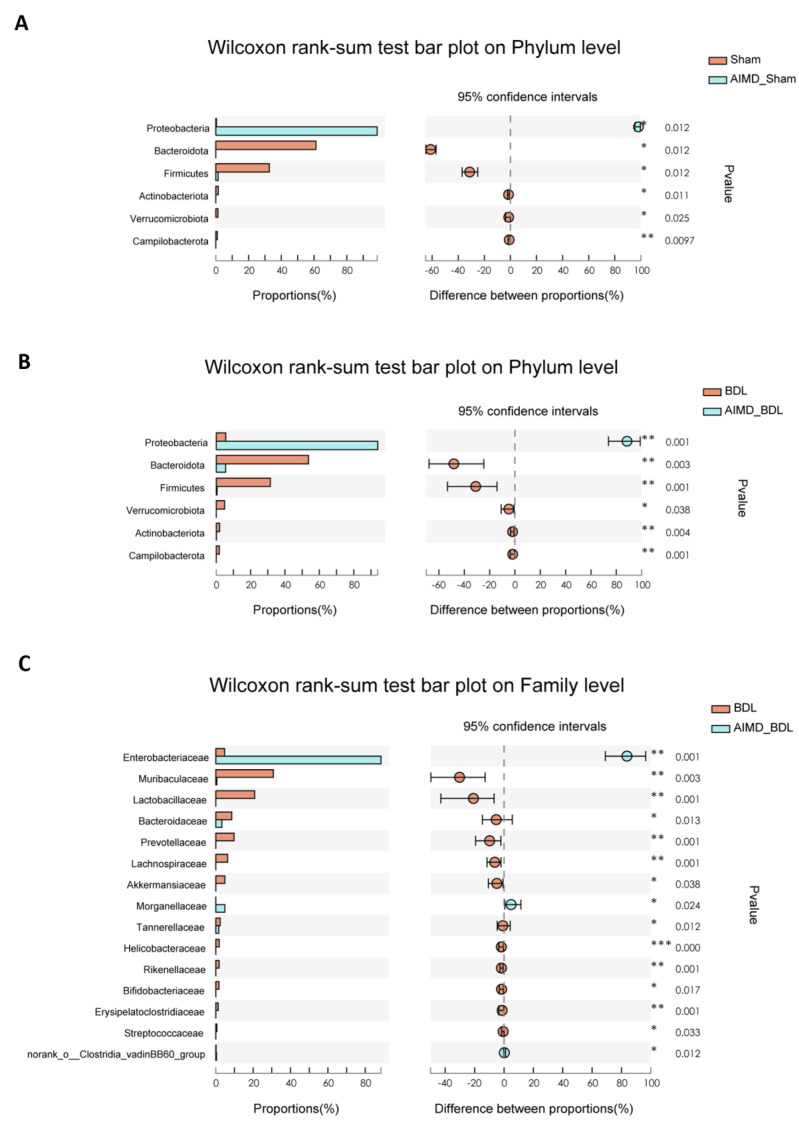
(**A**) Relative abundances of phylum responsible for discriminating the Sham group and AIMD_Sham group. (**B**) Relative abundances of families responsible for discriminating the BDL group and AIMD_BDL group. (**C**) Relative abundances of top 15 families responsible for discriminating the BDL group and AIMD_BDL group. Sham, *n* = 5; AIMD_Sham, *n* = 5; BDL, *n* = 7; AIMD_BDL groups, *n* = 8. * *p* < 0.05; ** *p* < 0.01; *** *p* < 0.001.

To evaluate the abundance and composition of the groups at different taxonomic levels, we plotted a histogram of community composition. At the phylum level, the vast majority of OTUs in each group belonged to five major phyla, including *Bacteroidetes*, *Firmicutes*, *Proteobacteria*, *Verrucomicrobi*, and *Actinobacteria* (Figure 2C). The microbiota composition of the BDL_AIMD group was similar to that of AIMD_Sham group, which indicated that AIMD drives the most substantial loss of diversity with BDL not appearing to compound upon that. Compared with the Sham group, the relative abundance of *Bacteroidetes*, *Firmicutes*, *Actinobacteria*, and *Verrucomicrobi* in the AIMD_Sham group was significantly lower (Figure 3A, *p* < 0.05). This result indicates that the use of antibiotics had a significant effect on the gut microbiota of mice, removing the originally dominant phylum.

At the family level, compared with the BDL group, the relative abundance of *Muribaculaceae* (*p* < 0.01), *Lactobacillaceae* (*p* < 0.01), *Bacteroidaceae* (*p* < 0.05), *Prevotellaceae* (*p* < 0.01), *Lachnospiraceae* (*p* < 0.01), *Akkermansiaceae* (*p* < 0.05), *Tannerellaceae* (*p* < 0.05), *Helicobacteraceae* (*p* < 0.001), *Rikenellaceae* (*p* < 0.01), *Bifidobacteriaceae* (*p* < 0.05), *Erysipelatoclostridiaceae* (*p* < 0.01), and *Streptococcaceae* (*p* < 0.05) were significantly decreased in the AIMD_BDL group, while *Enterobacteriaceae* (*p* < 0.01), *Morganellaceae* (*p* < 0.05), and *norank_o_Clostridia_vadinBB60_group* (*p* < 0.05) were significantly increased (Figure 3C). At the species level, the difference between the two groups was also significant (Figure S2). When comparing the BDL group with the Sham group at the family level (Figure S3), the relative abundance of *Erysipelotrichaceae* (*p* < 0.01), *Ruminococcaceae* (*p* < 0.05), *Desulfovibrionaceae* (*p* < 0.05), *unclassified_c__Bacilli* (*p* < 0.01), *Peptococcaceae* (*p* < 0.05), *norank_o__RF39* (*p* < 0.05), *Eubacterium_coprostanoligenes_group* (*p* < 0.01), *Butyricicoccaceae* (*p* < 0.05) were significantly lower in the BDL group.

### 2.3. AIMD_BDL Mice Harbor Different OTUs of Gut Microbiota versus BDL Mice

**Figure 4:**
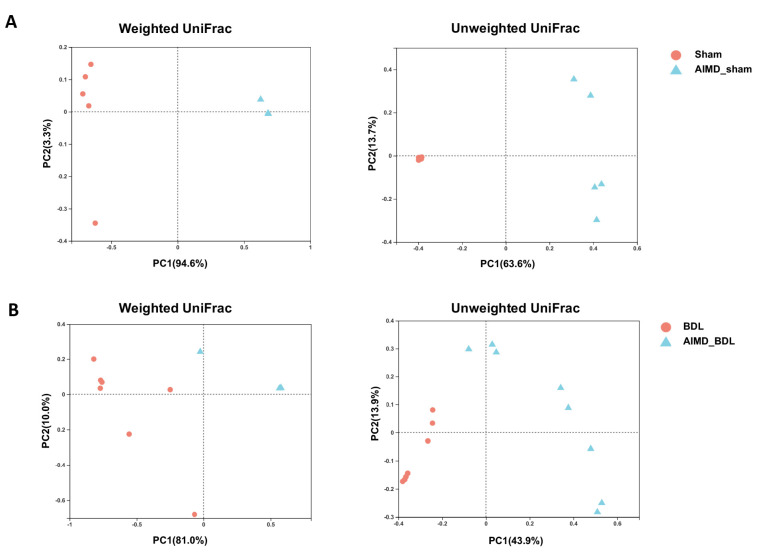
(**A**) Unweighted and weighted UniFrac distances showed obvious difference in gut microbiotic composition between Sham group (*n* = 5, red plots) and AIMD_Sham group (*n* = 5, blue plots). (**B**) Unweighted and weighted UniFrac distances showed obvious difference in gut microbiotic composition between BDL group (*n* = 7, red plots) and AIMD_BDL group (*n* = 8, blue plots).

To assess the differences in gut microbiota between the four groups, we performed PCoA analysis at the OTU level using the Weighted UniFrac distance and the Unweighted UniFrac distance algorithm. The Sham group could be clearly distinguished from the AIMD_Sham group, indicating that there were visible differences in the composition of the two groups, and the use of antibiotics affect the β-diversity of gut microbiota (Figure 4A). Using the Weighted UniFrac algorithm to analyze OTUs of the BDL group and the AIMD_BDL group, PC1 explained 81.0% of the variation, and PC2 explained 10.0% of the variation. Using the Unweighted UniFrac algorithm, PC1 explained 43.9% of the variation, and PC2 explained 13.9% of the variation (Figure 4B). These results suggest that the β-diversity of gut microbiota in the BDL group was significantly different from that of the AIMD_BDL group.

### 2.4. Gut Microbiota Deficiency Is Associated with Cholestatic Liver Injury

**Figure 5:**
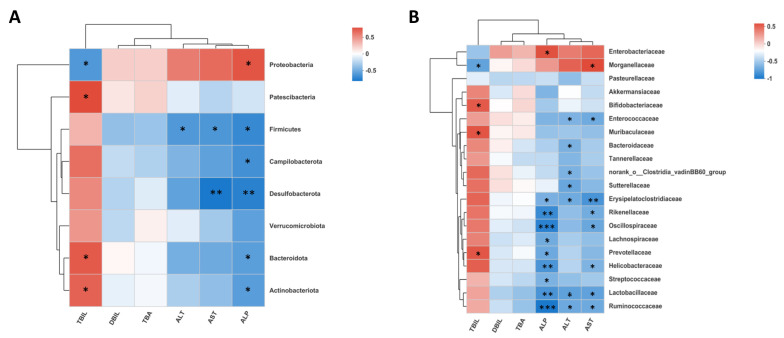
(**A**) The association between gut microbiota and serum markers of liver injury in AIMD_BDL group and BDL group at phylum level. (**B**) The association between gut microbiota and serum markers at family level. Red represents positive correlation, and blue represents negative correlation. * *p* < 0.05; ** *p* < 0.01; *** *p* < 0.001.

To investigate the relationship between gut microbiota and serum markers of cholestatic liver injury in mice, we performed a Spearman correlation analysis between gut microbiota and blood biochemical indices at the phylum, family, and genus levels, respectively. In the BDL group and AIMD_BDL group at the phylum level, *Proteobacteria* were negatively correlated with TBIL and positively correlated with ALP; *Patescibacteria* was positively correlated with TBIL; and *Firmicutes* was negatively correlated with ALT, AST, and ALP. *Campilobacterota* was negatively correlated with ALP, *Desulfobacterota* was negatively correlated with AST and ALP, and *Bacteroidota* and *Actinobacteriota* were positively correlated with TBIL and negatively correlated with ALP (Figure 5A). At the family level, *Lactobacillaceae*, *Ruminococcaceae*, *Erysipelatoclostridiaceae*, *Enterococcaceae*, *Sutterellaceae*, *norank_o_Clostridia_vadinBB60_ group*, and *Bacteroidaceae* were negatively correlated with ALT. *Lactobacillaceae*, *Ruminococcaceae*, *Erysipelatoclostridiaceae*, *Helicobacteraceae*, *Oscillospiraceae*, *Rikenellaceae*, *Enterococcaceae*, and *Morganellaceae* negatively correlated with AST. *Bifidobacteriaceae*, *Muribaculaceae*, *Morganellaceae*, and *Prevotellaceae* were significantly and positively correlated with TBIL (Figure 5B). At the genus level, *unclassified_ f_ Enterobacteriaceae* was positively correlated with AST and ALP and negatively correlated with TBIL; *Klebsiella* was positively correlated with AST and ALP; *Proteus* was positively correlated with AST and negatively correlated with TBIL; *Enterobacter* was negatively correlated with TBIL; *norank_ f_Lachnospiraceae*, *Alistipes*, *unclassified_f_Lachnospiracea*, *Erysipelatoclostridium*, and *Helicobacter* were negatively correlated with AST and ALP; *Escherichia-Shigella*, *norank_ f_ norank_ o_ Clostridia_ vadinBB60_ Group*, *Bacteroides*, and ALT were significantly negatively correlated; *Prevotellaceae_UCG-001* was negatively correlated with ALP; and *norank_ f_Muribaculateae*, *Bifidobacterium*, and TBIL were significantly positively correlated (Figure S4).

### 2.5. Gut Microbiota Deficiency Altered Gene Expression in the Livers of BDL Mice

**Figure 6:**
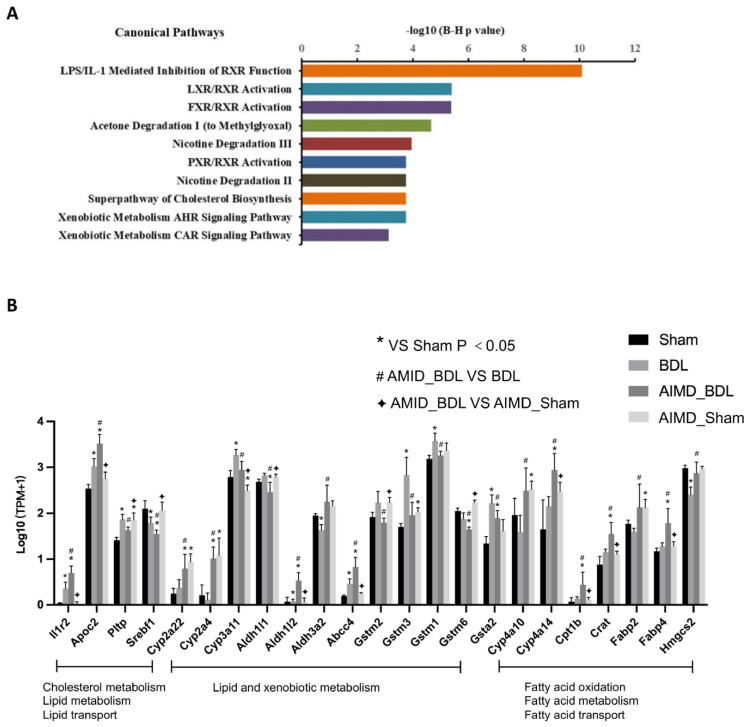
(**A**) Enriched IPA pathways of the DEGs between BDL group and AIMD_BDL group (−log10 (BH *p*-value), one−sided Fisher’s exact test). (**B**) The key molecules of the LPS/IL−1 Mediated Inhibition of RXR Function pathway in BDL mice, which mainly involved lipid and xenobiotic metabolism, fatty acid oxidation, and fatty acid transport. * *p* < 0.05 vs. Sham group; # *p* < 0.05 AIMD_BDL group vs. BDL group; ✦ *p* < 0.05 AIMD_BDL group vs. AIMD_Sham group.

To explore the causes and possible mechanisms of the exacerbation of cholestatic liver injury in gut microbiota deficiency mice, we performed liver transcriptome sequencing in the Sham, AIMD_Sham, BDL, and AIMD_BDL groups (five mice per group). We identified a total of 398 differential genes between the BDL group and AIMD_BDL group. The pathway analysis of differential genes used IPA software. We found that the main five signaling pathways involved in differential genes were LPS/IL-1 Mediated Inhibition of RXR Function, LXR/RXR Activation, FXR/RXR Activation, Acetone Degradation I (to Methylglyoxal), and Nicotine Degradation III (Figure 6A). The altered gut microbiota composition may affect key molecules of the LPS/IL-1 Mediated Inhibition of RXR Function pathway in BDL mice, so we analyzed the expression of genes involved in cholesterol, lipid metabolism, and lipid transport of the pathway (Figure 6B). Compared with the BDL group, Apoc2 was significantly higher in the AIMD_BDL group; Pltp and Srebf1 were significantly lower. Among the genes related to lipid and xenobiotic metabolism, Cyp2a22, Cyp2a4, Aldh1l2, Aldh3a2, and Abcc4 were significantly increased, and Cyp3a11, Aldh1l1, Gstm2, Gstm3, Gstm1, Gstm6, and Gsta2 were significantly decreased. Among the genes related to fatty acid transport, metabolism, and oxidation, Cyp4a10, Cyp4a14, Cpt1b, Crat, Fabp2, Fabp4, and Hmgcs2 were significantly highly expressed. We found that 14 genes involved in cholesterol, lipid metabolism, and lipid transport of the pathway were significantly different between AIMD_Sham and AIMD_BDL groups. Of these genes, only two differed between the Sham and AIMD_Sham groups (Figure 6B), which suggested that transcriptions of liver enzymes were not increased in AIMD-only treated mice.

**Figure 7:**
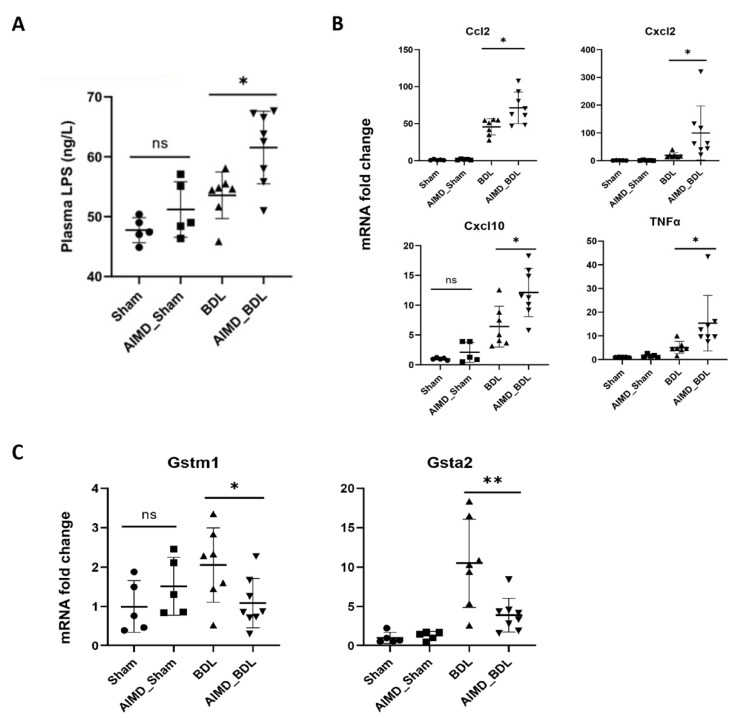
(**A**) The plasma levels of LPS in each group. (**B**) The expression of proinflammatory cytokines in the liver of each group. (**C**) The expression of glutathione transferases in the liver of each group. Sham, *n* = 5; AIMD_Sham, *n* = 5; BDL, *n* = 7; AIMD_BDL groups, *n* = 8. * *p <* 0.05; ** *p <* 0.01; ns, no significance.

Since 16S rRNA gene sequence analysis suggested that the intestinal microflora of mice in the AIMD_BDL group was dominated by the phylum *Proteobacteria* (Gram-negative bacteria), we examined the levels of LPS in the plasma of mice in each group. We found that the plasma levels of LPS were significantly higher in AIMD_BDL mice than in the rest of the groups (Figure 7A). Further, we examined the expression of proinflammatory cytokines in the livers of mice and found that the expression of TNFα, Ccl2, Cxcl2, and Cxcl10 was significantly higher in the AIMD_BDL group compared with the BDL group (Figure 7B). We also found that glutathione transferases Gstm1 and Gsta2, which protect liver function in the LPS/IL-1 Mediated Inhibition of RXR Function pathway, compensated by becoming increased in the BDL group, while significantly inhibited in the AIMD_BDL group (Figure 7C).

## 3. Discussion

AIMD models are widely used to explore the role of gut microbiota in various pathological situations [17], but there are no studies on mouse models of obstructive cholestasis in AIMD animals. Here, we report the effects of BDL surgery in AMID mice on serum markers and histological changes of liver injury, alterations in host gut microbiota composition, and on liver transcriptome. Our results suggest that BDL altered the composition of mice gut microbiota, and AIMD-induced alterations in gut microbiota are one of the factors that aggravate liver damage in BDL.

We observed that the abundance and diversity of the gut microbiota in mice after BDL was decreased, which is consistent with Cabrera-Rubio [18]. *Ruminococcaceae*, an important component of the microbiota of healthy individuals, maintains the balance of the intestinal microenvironment [19]. A gradual decrease in *Ruminococcaceae* abundance with increasing severity of liver fibrosis has also been observed in nonobese patients with nonalcoholic fatty liver disease (NAFLD) [20,21,22]. *F. prausnitzii*, which belongs to the family *Ruminococcaceae*, has been reported to modulate liver fat content and lipid species composition and reduce adipose tissue inflammation in high-fat fed mice [23]. Sinha et al. also reported that patients with ulcerative colitis after depletion of *Ruminococcaceae* showed increased colonic inflammation [24]. In our study, we found *Ruminococcaceae* was negatively correlated with ALT and AST. These results suggest the importance of *Ruminococcaceae* in maintaining the normal physiological function of gut microbiota.

The addition of antibiotics to drinking water in the AIMD model altered the composition structure, α-diversity, and β-diversity of gut microbiota. Both the AIMD_Sham and AIMD_BDL groups differed significantly from the Sham and BDL groups in α-diversity. Both the AIMD_Sham group and the AIMD_BDL group at the phylum level had *Proteobacteria* as the absolute dominant bacteria (more than 90%), which is consistent with the previous study [14]. Members of the phylum *Proteobacteria* are all Gram-negative bacteria, and in this study, the gut microbiota of both the AIMD_SHAM and AIMD_BDL groups were found to be dominated by *Klebsiella* spp. at the genus level. The abundance of *Proteobacteria* was elevated at 3 days postoperatively, probably due to a decrease in intestinal bile acid after bile duct ligation, which is more favorable for the growth of Gram-negative bacteria [25]. *Proteobacteria* have been shown to be positively associated with intestinal inflammation in previous studies [26,27]. Elevated *Proteobacteria* abundance upregulated LPS expression [28], while the levels of LPS, IL-6, IL-12, and TNF-α were downregulated when *Proteobacteria* abundance was reduced [29]. Thus, *Proteobacteria* may be involved in the LPS/IL-1 Mediated Inhibition of RXR Function pathway by regulating the expression of LPS and inflammatory factors. Correlation analysis revealed that *Klebsiella*, *unclassified_f__Enterobacteriaceae*, *Proteus*, and other Gram-negative genera with elevated abundance in the AIMD_BDL group were positively correlated with most serum markers of liver injury.

Based on the altered gut microbiota composition of the AIMD model with predominantly Gram-negative bacteria and the results of liver transcriptome analysis, we revealed that the inflammatory response induced by LPS in the AIMD_BDL group and its inhibition of glutathione S-transferase were important for the exacerbation of liver injury in the AIMD_BDL group. LPS is a major component of the outer membrane of Gram-negative bacteria, which can stimulate multiple signal cascades in immune and inflammatory cells (e.g., NF-kB, RK1/2, JNK mitogen-activated protein kinase, AP1), as well as stimulate the release of inflammatory factors (e.g., TNF, IL1α, IL1β, IL6, and IL10) [30,31,32], with important effects on liver injury, repair, and fibrosis. In addition, Choi et al. reported that the mRNA expression levels of rGSTA2, rGSTA3, rGSTM1, and rGSTM2 in mice livers were reduced when LPS was given intravenously [33]. We consider that the suppression of the glutathione transferase gene in AIMD_BDL mice is related to the fact that the dominant bacteria in this group is Gram-negative bacteria resulting in elevated levels of LPS and proinflammatory factors.

Another reason for the exacerbation of liver injury in the AIMD_BDL group may be related to the disturbance of lipid metabolism. According to the results of IPA analysis, differentially expressed genes were mainly enriched in lipid and xenobiotic metabolism, fatty acid oxidation, and fatty acid transport. For example, expression levels of Cyp4a10 and Cyp4a14 were higher in the AIMD_BDL group than in the BDL or Sham groups. The CYP4A subfamily is a cytochrome P450 fatty acid hydroxylase that catalyzes the ω-hydroxylation of medium- and long-chain fatty acids with prostaglandins [34]. CYP4A are partially affected by the Peroxisome proliferator-activated receptor-α. The current study shows that CYP4A increased production of hydrogen peroxide from long-chain fatty acid oxidation, which causes cellular damage and steatohepatitis [35]. In addition, we found that Cpt1b, a gene associated with fatty acid β-oxidation, was the most highly expressed in the AIMD_BDL group. Our study had limitations. The gut microbiota and derived microbial compounds are closely related to the metabolic mechanisms of the host. Therefore, future studies exploring the change of fecal metabolites can provide a supplement to our results.

In summary, we found that both BDL and AIMD led to a decrease in the abundance and diversity of gut microbiota in mice. Gut microbiota deficiency exacerbates liver injury in AIMD_BDL mice. This may be due to the predominance of Gram-negative bacteria in the gut microbiota of AIMD_BDL mice, resulting in elevated levels of LPS and proinflammatory factors, as well as inhibition of the expression of liver detoxification enzyme, leading to a disturbed lipid metabolism.

## 4. Materials and Methods

### 4.1. Animals

C57BL/6J male mice were obtained from the Experimental Animal Center of the Army Military Medical University, with 5–9 mice in each group. Mice were housed 3–4 per cage and maintained on a 12 h light/dark cycle at standard laboratory conditions (temperature 21 ± 1 °C, humidity 55 ± 5%) with free access to standard mouse chow and water. The experiment was approved by the animal ethics committee of the Army Medical University, and the guidelines for the management and use of laboratory animals were followed.

### 4.2. Antibiotic Pretreatment and Procedure of BDL in Mice

The 6-week-old C57BL/6J male mice were randomly divided into 4 groups. Group 1 and 2 both received a sham operation, but group 2 also was fed with antibiotic water to generate AIMD. Similarly, groups 3 and 4 both received BDL for 3 days, and group 4 was fed with antibiotic water to generate AIMD. The mixed antibiotics in the drinking water for AIMD groups contained: ampicillin 1 g/L, neomycin 1 g/L, metronidazole 1 g/L, and vancomycin 0.5 g/L [36]. The mixture of antibiotics was reconfigured, and the drinking water was changed every 3 days until the end of the experiment.

Three days after completion of the sham ligation and bile duct ligation procedures, the four groups of mice were weighed and euthanized. The livers and kidneys were weighed, and the blood, feces, liver, and large intestine of the mice were collected and stored in a deep cryogenic refrigerator or liquid nitrogen tank. The blood of mice was centrifuged, and the serum was aspirated and sent to the laboratory of our hospital to complete the blood biochemistry related indices by automatic biochemical instrument.

### 4.3. Hematoxylin-Eosin (HE) Staining and Liver Histology

The tissue blocks were paraffin-embedded and sliced. The slices were stained with hematoxylin and eosin. Liver histology was blindly assessed for necrosis on a 1 to 4+ scale.

### 4.4. Measurement of Lipopolysaccharide (LPS) Level in Plasma

The LPS concentrations were determined using an enzyme-linked immunosorbent assay (ELISA) kit, purchased from Jiangsu Meimian Industrial Co., Ltd. (Yancheng, China).

### 4.5. 16S rRNA Gene Sequence Analysis

Bacterial DNA was isolated from fecal samples using the E.Z.N.A.^®^ soil DNA Kit (Omega Bio-tek, Norcross, GA, USA). The V3-V4 region of the bacteria’s 16S rRNA gene was amplified by thermocycler PCR system (Thermo Fisher Scientific, Wilmington, USA). Amplicons were purified using the DNA Gel Extraction Kit (Axygen Biosciences, Union City, USA) and then were quantified. Purified amplicons were sequenced on an Illumina MiSeq platform (Illumina, San Diego, CA, USA). The 300 bp reads were truncated at any site receiving an average quality score <20. Quality control and splicing of raw data were performed using fastp (version 0.20.0) [37] and FLASH (version 1.2.7) [38]. Sequences with 97% similarity were clustered by operational taxonomic units (OTU) using UPARSE software (version 7.1) [39]. Each sequence was annotated with species classification using an RDP classifier (version 2.2) [40], and the Silva 16S rRNA database (version 138) was compared, with a 70% threshold. Alpha diversity analysis was used to evaluate the richness and diversity of the samples. The Good’s coverage index was used to reflect the sample coverage. Differences between groups were evaluated using Student’s *t*-test. Beta diversity was evaluated by unweighted and weighted UniFrac distance based on PCoA analysis.

### 4.6. RNA Sequencing

Total RNA from tissues was extracted using Trizol reagent (Invitrogen, Carlsbad, CA, USA) according to the manufacturer’s protocol. RNA quality was assessed with an Agilent 2100 bioanalyzer using RNA 6000 Nano Chip (Agilent, Santa Clara, CA, USA). The RNA-seq transcriptome library was prepared with the TruSeq^TM^ RNA sample preparation kit (Illumina, San Diego, CA, USA). The paired-end RNA-seq sequencing library was sequenced with the Illumina HiSeq 2000 (Illumina, San Diego, USA). The clean reads after quality control were compared with the reference genome using the HiSat2 (http://ccb.jhu.edu/software/hisat2/index.shtml, (accessed on 24 July 2020)) [41] and TopHat2 (http://ccb.jhu.edu/software/tophat/index.shtml, (accessed on 23 February 2016)) to obtain mapped reads. The gene expression levels were quantified using RSEM (http://deweylab.github.io/RSEM/, (accessed on 14 February 2020)) [42]. DESeq2 (http://bioconductor.org/packages/stats/bioc/DESeq2/ (accessed on 14 February 2020)) [43] were used to screen differentially expressed genes (DEG), which were defined as FDR < 0.05 and |log2FC| ≥ 1. Pathway analysis of DEGs was performed using Ingenuity Pathway Analysis (IPA, QIAGEN, Germantown, MD, USA).

### 4.7. Real-Time Quantitative Polymerase Chain Reaction (RT-PCR)

Total RNA was extracted from tissues with Trizol reagent (Invitrogen, Carlsbad, USA). Reverse transcription and real-time PCR analysis were performed with a cDNA synthesis kit (MBI Fermentas, Somerset, UK) and SYBR premix Ex Taq II kit (Takara Biotechnology, Mountain View, USA). RT-PCRs were analyzed with a Bio-Rad CFX96 (Bio-Rad, Berkeley, USA). The TaqMan Gene Expression Assays used were as follows: Gstm1 (Mm00833915_g1), Gsta2 (Mm03019257_g1), TNFα (Mm00443258_m1), Ccl2 (Mm00441242_m1), and Cxcl10 (Mm00445235_m1). The primers used were as follows: *cxcl2* 5′-aggcatctgcttcggggactctggc-3′ (forward) and 5′-gcaaactcagccacaggggcgaagg-3′ (reverse).

### 4.8. Statistical Analysis

All data are expressed as mean ± standard deviation (SD). Statistical analyses were performed using GraphPad Prism software (version 9.0, San Diego, USA) and SPSS (version 21, Chicago, USA). A Student’s *t*-test or Mann–Whitney U test was used to compare continuous variables. Categorical data were calculated with the Chi-square test or Fisher’s exact test. Correlations between two parameters were assessed using Spearman’s rank correlation coefficient. Significance was set as *p* < 0.05.

## Figures and Tables

**Table 1:** Serum markers of liver injury in each group after AIMD and BDL surgery.

	Sham	AIMD_Sham	BDL	AIMD_BDL
Subjects	*n* = 5	*n* = 5	*n* = 7	*n* = 8
ALT (IU/L)	17.28 ± 3.87	20.44 ± 3.26	365.49 ± 162.389 *	628.50 ± 256.92 *,#
AST (IU/L)	99.76 ± 10.91	109.60 ± 14.63	675.95 ± 328.53 *	1509.95 ± 565.15 *,#
ALP (IU/L)	116.00 ± 12.13	124.00 ± 21.91	413.71 ± 136.99 *	675.00 ± 242.84 *,#
TBA (μmol/L)	1.50 ± 1.06	1.88 ± 1.20	368.02 ± 211.81 *	449.65 ± 231.31 *
TBIL (μmol/L)	7.28 ± 4.65	5.68 ± 1.87	181.49 ± 42.73 *	139.46 ± 23.23 *,#
DBIL (μmol/L)	4.71 ± 3.28	3.91 ± 2.15	60.59 ± 29.62 *	84.48 ± 38.38 *

Sham, sham surgery; BDL, bile duct-ligated surgery; AIMD, antibiotic-induced microbiome depletion; ALT, alanine aminotransferase; AST, aspartate aminotransferase; ALP, alkaline phosphatase; TBA, total bile salts; TBIL, total bilirubin; DBIL, direct bilirubin. * *p* < 0.05 versus Sham mice; # *p* < 0.05 BDL versus AIMD_BDL mice.

## Data Availability

The datasets supporting the conclusions of this article are available in the Genome Sequence Archive (GSA ID CRA009058) with BioProject ID PRJCA013175 (Release date: 13 November 2024).

## Supplementary Materials

The following supporting information can be downloaded at: https://www.mdpi.com/article/10.3390/ijms24043180/s1.
