# Supplementary material for: Gut Microbiota Deficiency Exacerbates Liver Injury in Bile Duct Ligated Mice via Inflammation and Lipid Metabolism
Source: Int J Mol Sci. 2023 Feb 6;24(4):3180. doi: 10.3390/ijms24043180 (PMC9960910; doi:10.3390/ijms24043180)
Supplement: Supplementary file 1 [file ijms-24-03180-s001.zip › ijms-2124197-supplementary.pdf]

**Figure S1.**

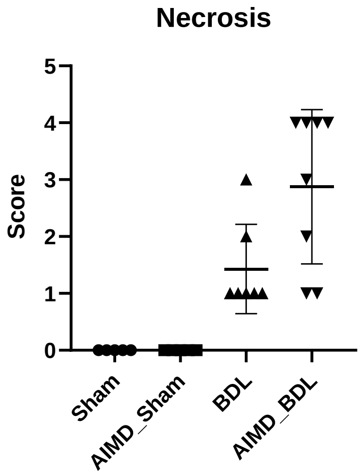


HE staining of liver tissues showed a significant higher scores for necrosis in the AIMD_BDL mice compared with the BDL mice.

**Figure S2.**

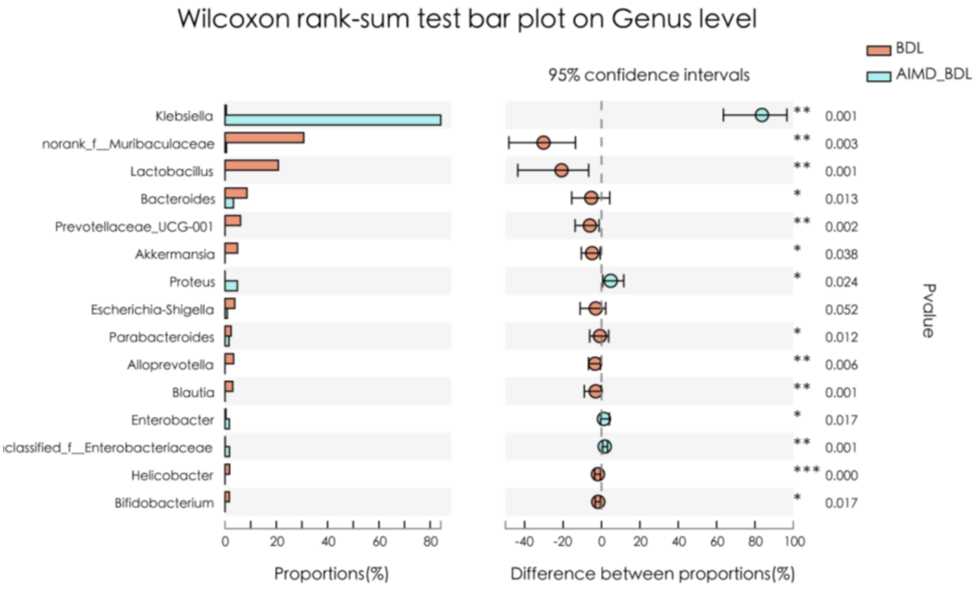


Relative abundances of top 15 genera responsible for discriminating the BDL and AIMD_BDL groups. BDL, n = 7; AIMD_BDL groups, n = 8. * P < 0.05 vs control; ** P < 0. 01; *** P < 0. 001.

**Figure S3.**

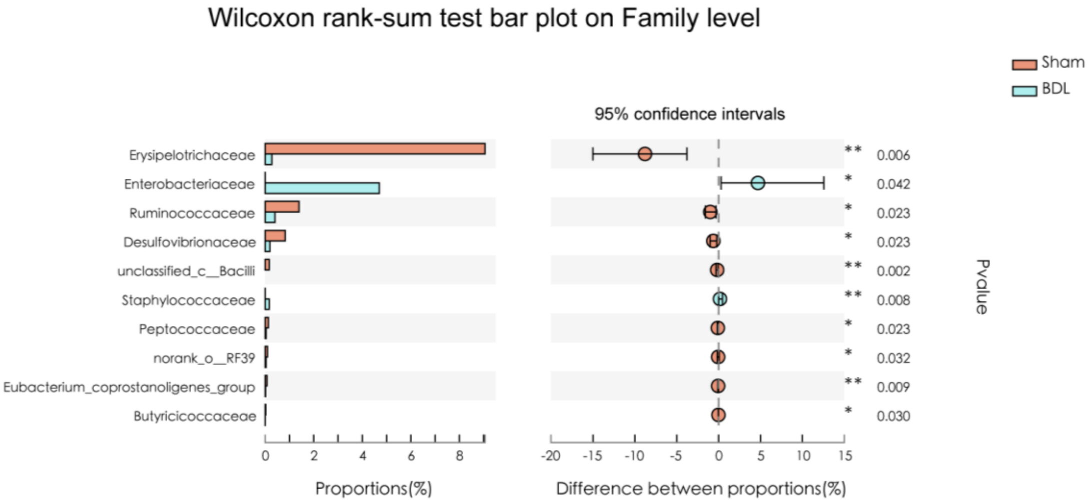


Relative abundances of top 10 families responsible for discriminating the BDL and Sham groups. BDL group, n = 7; Sham groups, n = 5. * P < 0.05 vs control; ** P < 0. 01.

**Figure S4.**

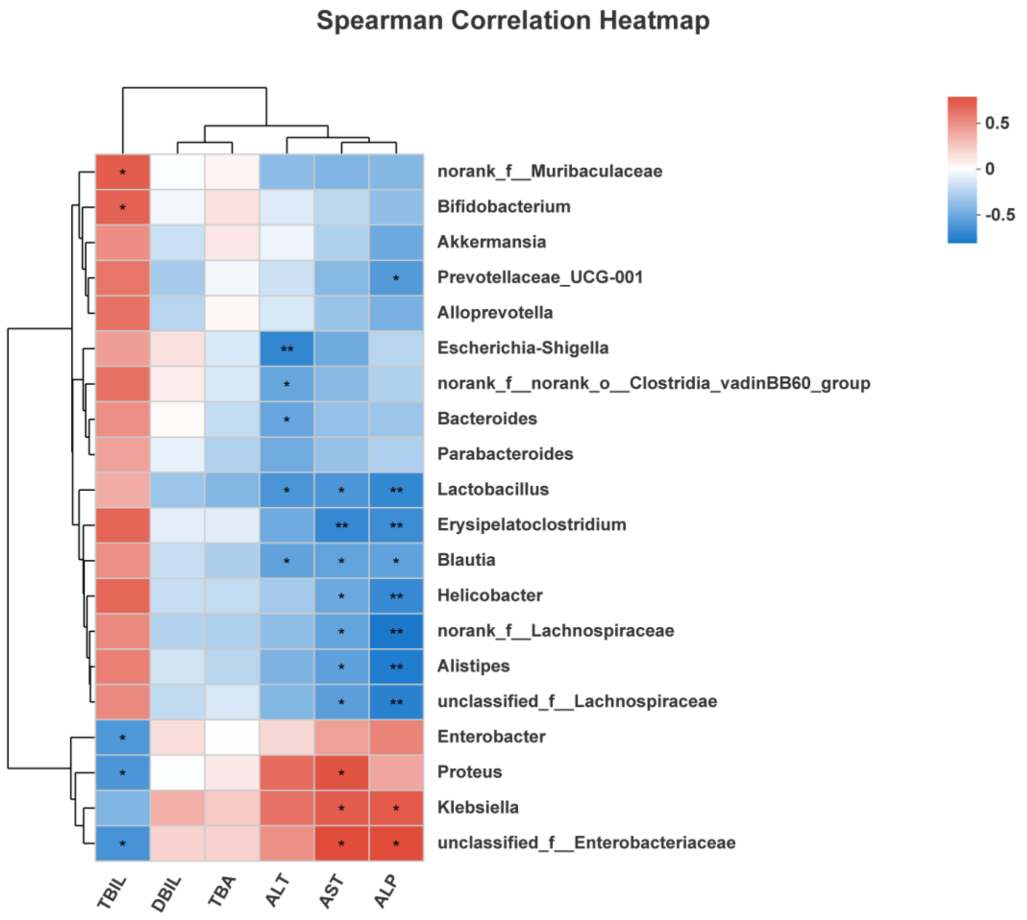


The association between gut microbiota and serum markers of liver injury in AIMD_BDL group and BDL group at genus level. Red represents positive correlation, and blue represents negative correlation. * P < 0.05; ** P < 0. 01; *** P < 0. 001.

**Table S1.** 16S RNA sequencing details for each sample.

| ID | Group name | Seq_num | Base_num | Mean_length | Min_length | Max_length |
|---|---|---|---|---|---|---|
| Sh1 | Sham | 55649 | 23486983 | 422.055796 | 332 | 511 |
| Sh2 | Sham | 51971 | 21897431 | 421.33942 | 277 | 494 |
| Sh3 | Sham | 47703 | 20095016 | 421.252668 | 248 | 496 |
| Sh4 | Sham | 53288 | 22569785 | 423.543481 | 262 | 438 |
| Sh5 | Sham | 64638 | 27091437 | 419.125545 | 280 | 483 |
| ASh1 | AIMD_sham | 36566 | 15684610 | 428.939725 | 403 | 430 |
| ASh2 | AIMD_sham | 40671 | 17447275 | 428.985641 | 404 | 454 |
| ASh3 | AIMD_sham | 45508 | 19522319 | 428.98653 | 404 | 472 |
| ASh4 | AIMD_sham | 40207 | 17248339 | 428.98846 | 404 | 478 |
| ASh5 | AIMD_sham | 41546 | 17820449 | 428.932966 | 308 | 431 |
| B1 | BDL | 38171 | 16096295 | 421.68911 | 345 | 444 |
| B2 | BDL | 61252 | 25863295 | 422.24409 | 265 | 430 |
| B3 | BDL | 52977 | 22242598 | 419.853861 | 252 | 444 |
| B4 | BDL | 37707 | 16146914 | 428.220596 | 277 | 477 |
| B5 | BDL | 56101 | 23632010 | 421.240441 | 258 | 473 |
| B6 | BDL | 46688 | 19725582 | 422.497901 | 277 | 444 |
| B7 | BDL | 56287 | 23768281 | 422.269458 | 325 | 445 |
| AB1 | AIMD_BDL | 61044 | 26187085 | 428.987042 | 404 | 430 |
| AB2 | AIMD_BDL | 36575 | 15573043 | 425.783814 | 333 | 444 |
| AB3 | AIMD_BDL | 37909 | 16262252 | 428.981297 | 371 | 503 |
| AB4 | AIMD_BDL | 38416 | 16479933 | 428.986178 | 404 | 430 |
| AB5 | AIMD_BDL | 44843 | 19237073 | 428.9872 | 404 | 431 |
| AB6 | AIMD_BDL | 38497 | 16514273 | 428.975583 | 282 | 430 |
| AB7 | AIMD_BDL | 41269 | 17701606 | 428.932274 | 403 | 517 |
| AB8 | AIMD_BDL | 38054 | 16317937 | 428.810033 | 251 | 431 |

Sham, sham surgery; BDL, bile duct-ligated surgery; AIMD, Antibiotic-induced microbiome depletion.

**Table S2.** Alpha diversity for each sample.

| ID | Group | sobs | shannon | simpson | ace | chao | invsimpson | coverage |
|---|---|---|---|---|---|---|---|---|
| Sh1 | Sham | 392 | 4.0801 | 0.0364 | 442.5732 | 447.5610 | 27.4894 | 0.9977 |
| Sh2 | Sham | 377 | 4.0832 | 0.0336 | 416.7544 | 420.8718 | 29.7997 | 0.9980 |
| Sh3 | Sham | 346 | 3.7978 | 0.0665 | 392.6966 | 427.3750 | 15.0372 | 0.9979 |
| Sh4 | Sham | 336 | 3.7310 | 0.0521 | 388.6073 | 407.5000 | 19.2063 | 0.9978 |
| Sh5 | Sham | 285 | 2.6387 | 0.2256 | 331.2086 | 342.0000 | 4.4318 | 0.9981 |
| ASh1 | AIMD_sham | 32 | 0.1455 | 0.9565 | 85.1375 | 67.0000 | 1.0455 | 0.9995 |
| ASh2 | AIMD_sham | 17 | 0.1725 | 0.9415 | 177.9428 | 72.0000 | 1.0622 | 0.9996 |
| ASh3 | AIMD_sham | 19 | 0.1027 | 0.9697 | 117.3406 | 74.0000 | 1.0313 | 0.9996 |
| ASh4 | AIMD_sham | 14 | 0.3542 | 0.8466 | 22.7500 | 17.3333 | 1.1812 | 0.9998 |
| ASh5 | AIMD_sham | 19 | 0.1580 | 0.9527 | 25.7552 | 21.0000 | 1.0497 | 0.9999 |
| B1 | BDL | 154 | 2.6791 | 0.1328 | 188.8539 | 217.0000 | 7.5321 | 0.9988 |
| B2 | BDL | 148 | 3.1352 | 0.0786 | 169.3606 | 180.5000 | 12.7250 | 0.9991 |
| B3 | BDL | 277 | 3.3344 | 0.0791 | 307.3671 | 304.9730 | 12.6369 | 0.9985 |
| B4 | BDL | 208 | 1.6315 | 0.3578 | 244.2514 | 237.2162 | 2.7945 | 0.9984 |
| B5 | BDL | 240 | 3.0548 | 0.1178 | 281.5917 | 274.8710 | 8.4902 | 0.9984 |
| B6 | BDL | 268 | 3.0984 | 0.1138 | 301.2110 | 322.0500 | 8.7890 | 0.9984 |
| B7 | BDL | 263 | 3.1818 | 0.1169 | 282.5387 | 281.3704 | 8.5568 | 0.9989 |
| AB1 | AIMD_BDL | 10 | 0.1441 | 0.9529 | 13.6857 | 11.5000 | 1.0495 | 0.9999 |
| AB2 | AIMD_BDL | 61 | 2.0114 | 0.1847 | 63.8895 | 62.6667 | 5.4132 | 0.9998 |
| AB3 | AIMD_BDL | 17 | 0.0673 | 0.9799 | 302.4341 | 50.0000 | 1.0205 | 0.9996 |
| AB4 | AIMD_BDL | 8 | 0.0837 | 0.9750 | 11.6667 | 9.0000 | 1.0256 | 0.9999 |
| AB5 | AIMD_BDL | 13 | 0.2396 | 0.9146 | 34.2342 | 23.0000 | 1.0933 | 0.9998 |
| AB6 | AIMD_BDL | 18 | 0.2000 | 0.9301 | 23.5000 | 21.7500 | 1.0752 | 0.9998 |
| AB7 | AIMD_BDL | 57 | 0.8857 | 0.5104 | 124.1668 | 114.2727 | 1.9591 | 0.9988 |
| AB8 | AIMD_BDL | 94 | 0.4515 | 0.8324 | 140.9383 | 131.0500 | 1.2013 | 0.9987 |

Sham, sham surgery; BDL, bile duct-ligated surgery; AIMD, Antibiotic-induced microbiome depletion.
